# IGF2BP2 contributes to thyroid cancer progression by enhancing the stability of m6A-modified CTSH mRNA

**DOI:** 10.1371/journal.pone.0332061

**Published:** 2025-10-16

**Authors:** Liangpeng Dong, Lingyun Chen, Xiaofen Qi, Qiaochu Lu, Youwei Li, Mengqi Hou

**Affiliations:** 1 Department of Thyroid and Breast Surgery, The First Affiliated Hospital of Xinxiang Medical University, Weihui, Henan, China; 2 Operating room, the First Affiliated Hospital of Xinxiang Medical University, Weihui, Henan, China; Xiangya Hospital Central South University, CHINA

## Abstract

**Background:**

N6-methyladenosine (m6A) is a prevalent RNA modification in eukaryotes that regulates RNA stability and translation. Dysregulated m6A modification is implicated in cancer progression. This study investigated the role of the m6A reader protein, insulin-like growth factor 2 mRNA-binding protein 2 (IGF2 BP2), in the progression of thyroid cancer (TC).

**Methods:**

Cell proliferation was assessed using cell counting kit-8 (CCK8) and 5-ethynyl-2’-deoxyuridine (EdU) assays. Cell migration and invasion were evaluated by Transwell assays. A xenograft tumor model was employed to examine the impact of IGF2 BP2 on tumor growth *in vivo*. Gene functional annotation was performed through GO analysis. Spearman correlation analysis was utilized to evaluate the relationship between the expression levels of cathepsin H (CTSH) and IGF2 BP2. RIP-qPCR and RNA pull-down assays were conducted to confirm the interaction between IGF2 BP2 and CTSH mRNA.

**Results:**

Elevated IGF2 BP2 expression correlated significantly with advanced N stage in TC. Knockdown of IGF2 BP2 inhibited TC cell proliferation, migration, and invasion *in vitro*, as well as tumor growth *in vivo*. CTSH expression mirrored IGF2 BP2 expression. IGF2 BP2 interacted with CTSH mRNA, enhancing its stability in an m6A-dependent manner. Overexpression of CTSH counteracted the effects of IGF2 BP2 knockdown on TC cell proliferation, migration, invasion, and epithelial-to-mesenchymal transition (EMT).

**Conclusion:**

IGF2 BP2 accelerates TC progression by recognizing and stabilizing m6A-modified CTSH mRNA. IGF2 BP2 and CTSH represent potential diagnostic and therapeutic targets for TC.

## Introduction

Thyroid cancer (TC), originating from thyroid follicular epithelial or parafollicular cells, represents the most common malignancy of the endocrine system, with an increasing incidence globally [1]. The rapid rise in TC cases is attributed to several factors, including advances in cross-sectional imaging, increased exposure to ionizing radiation, obesity, environmental influences, and smoking-related endocrine disorders [2]. While the majority of patients with TC have a favorable prognosis, 10%−20% experience poor outcomes due to recurrence, distant metastasis, drug resistance, or local invasion [3,4]. Thus, further molecular research is urgently needed to unravel the pathogenesis of TC.

Since the 1960s, over 170 post-transcriptional RNA modifications have been identified [5]. However, the biological significance of RNA methylation has only been gradually understood in the past decade [6]. N6-methyladenosine (m6A) is a prevalent modification in eukaryotic messenger RNA (mRNA) that plays a critical role in regulating RNA processing, splicing, translation, and stability. m6A modification is a reversible process, regulated by “writer” methylases and “eraser” demethylases. Additionally, m6A-modified sites are recognized by “reader” proteins, such as insulin-like growth factor 2 mRNA-binding proteins (IGF2 BPs). [7] The interplay of these proteins—writers, erasers, and readers—determines m6A modification’s impact on biological processes by catalyzing, removing, or interpreting m6A marks.

Increasing evidence highlights the role of m6A modification in various cancers [8], including acute myeloid leukemia, glioblastoma, lung cancer, and liver cancer. In papillary thyroid cancer (PTC), Zhu *et al*. demonstrated that METTL3-mediated m6A modification of STEAP2 mRNA suppressed PTC progression by affecting the Hedgehog signaling pathway and epithelial-to-mesenchymal transition (EMT). [9] IGF2 BP2, a member of the IGF2 BP family, is a key “reader” protein involved in m6A modification. Overexpression of IGF2 BP2 in human cancers has been linked to poor prognosis [10–12]. Our previous study identified IGF2 BP2 as aberrantly upregulated in TC, where it positively regulated HAGLR expression in an m6A-dependent manner, contributing to TC progression [13]. The present study further investigated the mechanisms underlying IGF2 BP2’s role in TC.

In this study, the biological function of IGF2 BP2 in TC was validated both *in vitro* and *in vivo*. Subsequently, potential downstream targets of IGF2 BP2 were explored to elucidate its mechanistic role in TC.

## Results

### IGF2 BP2 knockdown restrains BCPAP cell proliferation, migration, and invasion *in vitro*

**Fig 1:**
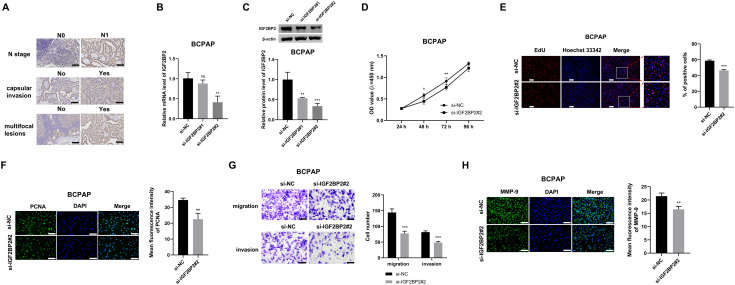
IGF2 BP2 knockdown inhibits BCPAP cell proliferation, migration, and invasion *in vitro.* **(A)** IHC analysis of IGF2 BP2 protein localization and relative abundance in samples of patients with PTC. Representative images illustrate differential IGF2 BP2 staining intensity in tumors categorized by N stage (N0 vs N1), capsular invasion status (No vs Yes), or multifocal lesions (No vs Yes). Scale bars: 100 μm. **(B)** RT-qPCR analysis of the interference efficiencies of two IGF2 BP2-specific siRNAs. **(C)** Western blot validation of the knockdown efficiency of IGF2 BP2-specific siRNAs. **(D-H)** BCPAP cells were transfected with either si-NC or si-IGF2 BP2#2. **(D and E)** CCK8 and EdU assays were used to analyze cell proliferation ability. Scale bars: 200 μm. **(F)** PCNA expression was measured by IF assay. Scale bars: 100 μm. **(G)** Transwell assays were conducted to assess cell migration and invasion. Scale bars: 100 μm. **(H)** MMP9 protein levels were analyzed by IF assay. Scale bars: 100 μm. ^*^P < 0.05, ^**^P < 0.01, ^***^P < 0.001, ns: not statistically significant. IGF2 BP2: insulin-like growth factor 2 mRNA binding protein 2; EdU: 5-ethynyl-2’-deoxyuridine; PCNA: proliferating cell nuclear antigen; MMP-9: matrix metalloproteinase 9.

To assess the clinical relevance of IGF2 BP2 in PTC, data from 497 PTC samples were obtained from the TCGA database. Clinical information on metastasis (M), regional lymph node (N), tumor (T), and tumor malignancy degree were available for 289, 449, 495, and 495 samples, respectively. Based on the median IGF2 BP2 expression, the samples were divided into high and low expression groups, and correlations between IGF2 BP2 expression and tumor M, N, T stage, and malignancy degree were analyzed. The results indicated that elevated IGF2 BP2 expression was significantly associated with advanced N stage, highlighting its potential involvement in lymph node metastasis, a critical factor in cancer progression and prognosis. However, no significant correlation was found between IGF2 BP2 levels and M, T, or tumor malignancy degree (**Table 1**). Furthermore, IGF2 BP2 protein localization and relative abundance were assessed in PTC samples *via* immunohistochemistry (IHC), with representative images stratified by clinicopathological features shown in **Fig 1A**. The results demonstrated that IGF2 BP2 was upregulated in samples with N1 stage, capsular invasion, and multifocal lesions, indicating its critical role in PTC progression through its impact on tumor aggressiveness and dissemination. In line with our previous study, [13] IGF2 BP2 knockdown suppressed TPC-1 cell proliferation, cell cycle progression, migration, invasion, and induced apoptosis. To further validate these findings in a cellular model, loss-of-function experiments were performed in the BCPAP TC cell line. Transfection with si-IGF2 BP2#2 significantly reduced IGF2 BP2 mRNA and protein expression (**Fig 1B**, C). Given its significant knockdown effect, si-IGF2 BP2#2 was selected for further analysis, whereas si-IGF2 BP2#1 was ineffective. CCK8 and EdU assays demonstrated that IGF2 BP2 knockdown inhibited BCPAP cell proliferation (**Fig 1D**, E). As PCNA is a well-known marker of cell proliferation, immunofluorescence (IF) assays revealed a reduction in PCNA levels in BCPAP cells upon IGF2 BP2 knockdown (**Fig 1F**). Transwell assays showed a marked decrease in the number of migrated and invaded cells (**Fig 1G**), suggesting that IGF2 BP2 knockdown inhibited BCPAP cell migration and invasion, both of which are critical for cancer metastasis. MMP-9, a key protein involved in tumor metastasis, was also downregulated in BCPAP cells following IGF2 BP2 knockdown, as shown by IF assays (**Fig 1H**).

**Table 1:** Correlations between IGF2BP2 expression and clinicopathologic features.

Phenotype	Case	IGF2BP2 expression		*P*-value
		High Low		
Pathologic_M				0.069908714
M0	282	144	138	
M1	7	6	1	
Pathologic_N				0.000192992^***^
N0	224	93	131	
N1	225	133	92	
Pathologic_T				0.129967007
T1&T1	305	144	161	
T3&T4	190	103	87	
Stage				0.372255849
I&II	332	161	171	
III&IV	163	86	77	
Gender				0.861150104
Male	134	66	68	
Female	363	182	181	

***P＜0.001

To confirm the specificity of IGF2 BP2 knockdown, an siRNA-resistant IGF2 BP2 mutant (IGF2 BP2-MUT) was engineered, incorporating silent mutations at the siRNA#2 binding site. While IGF2 BP2-WT failed to restore expression under siRNA#2 knockdown, IGF2 BP2-MUT successfully rescued both mRNA and protein levels (S1A,B Fig). Functionally, IGF2 BP2-MUT reversed the anti-proliferative effects of siRNA#2 in CCK-8 assay (S1C Fig) and restored migration and invasion capacities (S1D Fig). These rescue experiments confirm the on-target specificity of IGF2 BP2 knockdown. Collectively, these results support the role of IGF2 BP2 as an oncogene in TC, promoting cell proliferation, migration, and invasion, thereby contributing to tumor progression and potential metastasis.

### IGF2 BP2 interference impairs xenograft tumor growth *in vivo*

**Fig 2:**
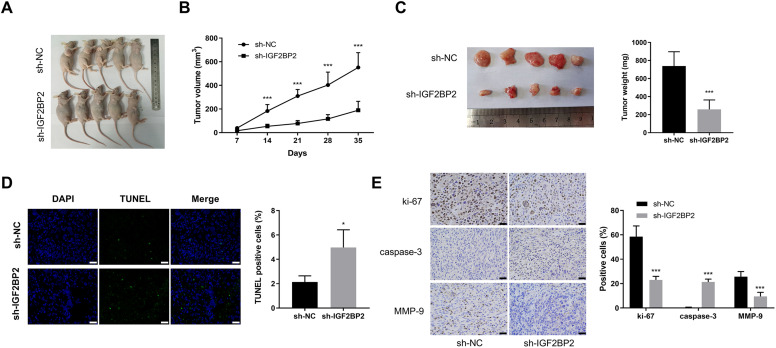
IGF2 BP2 interference impairs xenograft tumor growth *in vivo.* **(A)** Photographs of xenograft tumor formation in nude mice. **(B)** Tumor volume was measured at the indicated time points. **(C)** Tumor mass was recorded after tumor excision at day 35. **(D)** TUNEL staining was performed to assess tumor cell apoptosis. Scale bars: 200 μm. **(E)** IHC assay was conducted to evaluate the protein localization and relative abundance of proliferation marker Ki-67, apoptosis marker caspase-3, and metastasis marker MMP-9 in resected tumor tissues from both groups. Scale bars: 50 μm. ^*^P < 0.05, ^***^P < 0.001. IGF2 BP2: insulin-like growth factor 2 mRNA binding protein 2; TUNEL: terminal deoxynucleotidyl transferase-mediated dUTP nick end labeling; MMP-9: matrix metalloproteinase 9.

To further validate the role of IGF2 BP2 in TC *in vivo*, a subcutaneous xenograft tumor model was established in nude mice. Representative images of xenograft tumor formation are shown in **Fig 2A**. Tumor volume was measured every 7 days, revealing that IGF2 BP2 knockdown significantly impaired xenograft tumor growth compared to control groups (**Fig 2B**). At 35 days post-inoculation, the mice were euthanized, and xenograft tumors were excised for mass measurement. Tumors from IGF2 BP2-knockdown cell inoculations exhibited significantly reduced mass compared to the sh-NC group (**Fig 2C**). TUNEL assay demonstrated a marked increase in green fluorescent points in the IGF2 BP2 knockdown group, indicating enhanced tumor cell apoptosis (**Fig 2D**). IHC assay results showed that IGF2 BP2 knockdown led to a decrease in the relative abundance of Ki-67 and MMP-9, while upregulating the relative abundance of caspase-3 in tumor tissues (**Fig 2E**). These changes suggest anti-proliferative and pro-apoptotic effects, consistent with the *in vitro* results. These findings highlight the oncogenic role of IGF2 BP2, highlighting its involvement in tumor proliferation, apoptosis resistance, and metastatic potential in TC.

### CTSH is a potential target of RBP IGF2 BP2

**Fig 3:**
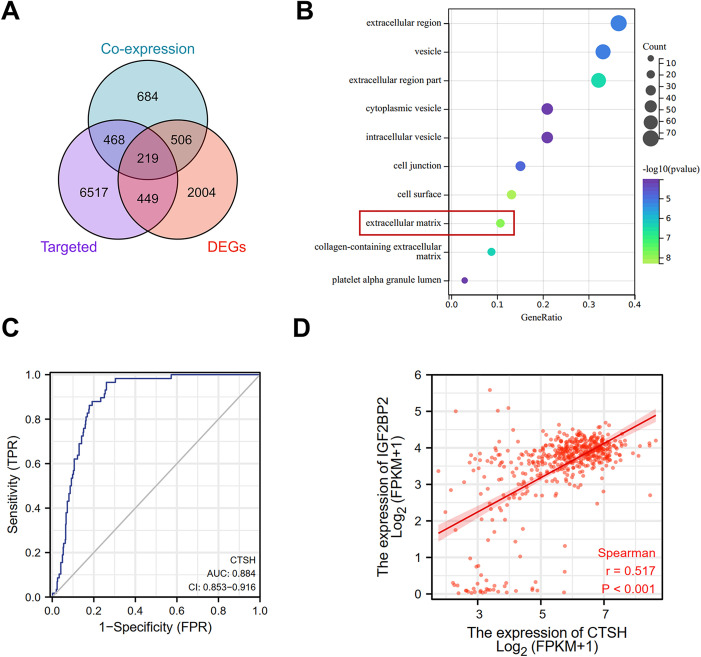
CTSH is a potential target of RBP IGF2 BP2. **(A)** m6A2Target database was used to predict potential targets of RBP IGF2 BP2. TCGA-THCA data were analyzed to identify genes co-expressed with IGF2 BP2. Venn diagram showing 219 overlapping genes across three groups. **(B)** The 219 overlapping genes underwent GO cellular component annotation. **(C)** ROC curve was plotted to evaluate the diagnostic value of CTSH levels in TC. **(D)** Spearman correlation analysis was used to assess the linear correlation between CTSH and IGF2 BP2 expression. IGF2 BP2: insulin-like growth factor 2 mRNA binding protein 2; CTSH: cathepsin **H.**

IGF2 BP2, as an m6A reader protein, recognizes modified m6A sites on mRNAs and enhances the stability of target mRNAs. To explore the molecular mechanisms underlying its oncogenic function, potential downstream mRNA targets of IGF2 BP2 were investigated. The m6A2Target database was used to predict IGF2 BP2’s possible targets, and genes co-expressed with IGF2 BP2 were identified through TCGA-THCA data. Differentially expressed genes (DEGs) between PTC and normal samples were also analyzed. The gene data across these three groups is presented in S1 Table. A Venn diagram illustrated 219 overlapping genes from the three groups (**Fig 3A**). Functional annotation of these overlapping genes was performed using Gene Ontology (GO) analysis (S2 Table). GO-Cellular Component (CC) analysis revealed significant enrichment in extracellular matrix components (**Fig 3B**). Among these, 22 genes were extracted for further analysis, focusing on those most closely associated with TC growth and progression. CTSH expression has been previously reported to be notably elevated in TC, [14] and our analysis showed that high CTSH expression correlated with higher N stage (**Table 2**). Additionally, receiver operating characteristic (ROC) curve analysis indicated that CTSH exhibited high diagnostic value for TC, with an area under the curve (AUC) of 0.884 (**Fig 3C**). Spearman correlation analysis revealed a significant positive correlation between CTSH and IGF2 BP2 expression (*r* = 0.517, *P* < 0.001) (**Fig 3D**), suggesting that CTSH may be a molecular target of IGF2 BP2.

**Table 2:** Correlations between CTSH expression and clinicopathologic features.

Characteristic	CTSH expression		*P* value	Statistical value	Method
	Low	High			
*n*	255	255			
T stage, n (%)			0.072	6.99	Chisq.test
T1	78 (15.4%)	65 (12.8%)			
T2	90 (17.7%)	77 (15.2%)			
T3	80 (15.7%)	95 (18.7%)			
T4	7 (1.4%)	16 (3.1%)			
N stage, n (%)			<0.001	22.61	Chisq.test
N0	137 (29.8%)	92 (20%)			
N1	86 (18.7%)	145 (31.5%)			
M stage, n (%)			1.000		Fisher.test
M0	129 (43.7%)	157 (53.2%)			
M1	4 (1.4%)	5 (1.7%)			
Age, median (IQR)	46 (34, 60)	47 (36, 57)	0.844	32841	Wilcoxon

### IGF2 BP2 interacts with CTSH mRNA

**Fig 4:**
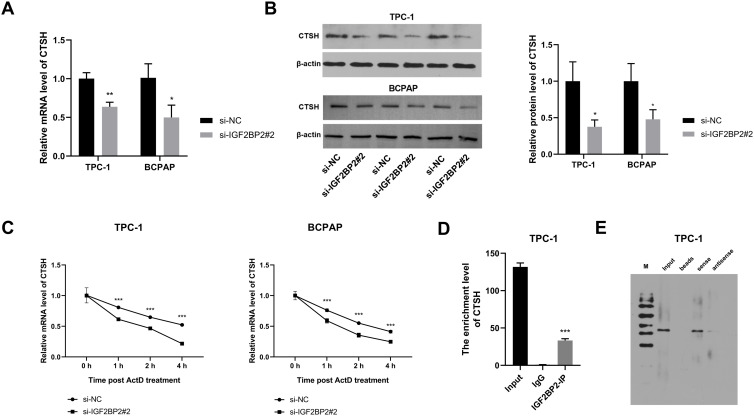
IGF2 BP2 interacts with CTSH mRNA. **(A and B)** TPC-1 and BCPAP cells were transfected with si-NC or si-IGF2 BP2#2, and the mRNA and protein expression of CTSH were analyzed by RT-qPCR and Western blot. **(C)** The stability of CTSH mRNA with or without IGF2 BP2 knockdown was assessed through actinomycin D treatment. **(D)** RIP-qPCR was performed to verify the interaction between IGF2 BP2 and CTSH mRNA. **(E)** RNA pull-down assay using biotin-labeled CTSH probe was conducted to confirm the binding relationship between CTSH mRNA and IGF2 BP2. The IGF2 BP2 level in the bead-probe-protein complex was analyzed by Western blot. ^*^P < 0.05, ^**^P < 0.01, ^***^P < 0.001, ns: not statistically significant. IGF2 BP2: insulin-like growth factor 2 mRNA binding protein 2; CTSH: cathepsin **H.**

To investigate the regulatory interaction between IGF2 BP2 and CTSH mRNA, the potential role of IGF2 BP2 in regulating CTSH mRNA stability and its subsequent impact on CTSH mRNA and protein levels was explored. IGF2 BP2 knockdown led to a reduction in both mRNA and protein levels of CTSH in two TC cell lines (**Fig 4A**, B). To assess the effect of IGF2 BP2 on CTSH mRNA stability, ActD was applied, revealing a significant increase in RNA decay rates following IGF2 BP2 knockdown (**Fig 4C**). RIP-qPCR assays demonstrated a significant enrichment of CTSH when using the IGF2 BP2 antibody (**Fig 4D**), indicating direct binding between IGF2 BP2 and CTSH mRNA. The Starbase database was employed to predict the binding domain between CTSH mRNA and IGF2 BP2, and a 200 bp biotin-labeled probe corresponding to this domain was synthesized. RNA pull-down assays were then performed to further confirm the interaction between CTSH mRNA and IGF2 BP2 using the biotin-labeled probe, with an antisense probe serving as a control. The data revealed significant enrichment of IGF2 BP2 protein in the sense probe group, while no enrichment was observed in the antisense probe group (**Fig 4E**). These findings unveil a novel molecular interaction in which IGF2BP2 regulates CTSH mRNA stability and expression, contributing to their cooperative role in TC pathophysiology.

### CTSH overexpression partly abolishes IGF2BP2 knockdown-mediated anti-proliferative effect in TPC-1 and BCPAP cells

**Fig 5:**
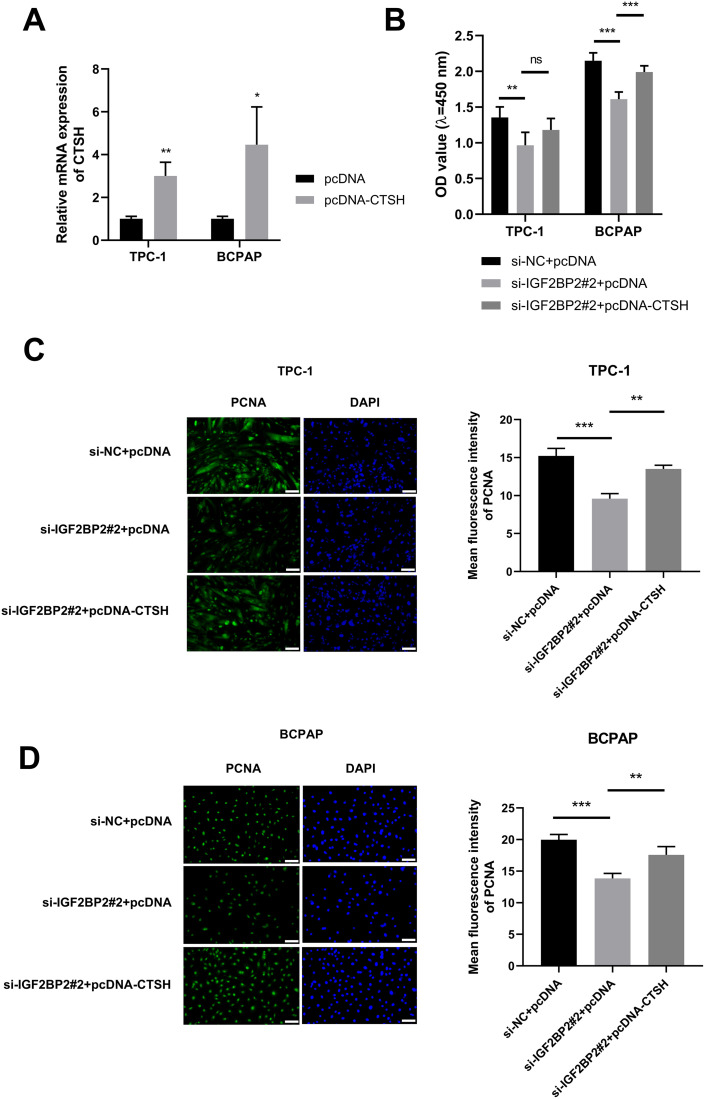
CTSH overexpression partially reverses IGF2 BP2 knockdown-mediated anti-proliferative effects in TPC-1 and BCPAP cells. **(A)** Overexpression efficiency of the CTSH plasmid in TPC-1 and BCPAP cells was analyzed by RT-qPCR. **(B-D)** TPC-1 and BCPAP cells were divided into three groups: si-NC + pcDNA, si-IGF2 BP2#2 + pcDNA, and si-IGF2 BP2#2 + pcDNA-CTSH. **(B)** CCK8 assay was performed to assess cell viability. **(C and D)** Protein expression of PCNA was measured by IF assay. Scale bars: 100 μm. ^*^P < 0.05, ^**^P < 0.01, ^***^P < 0.001, ns: not statistically significant. IGF2 BP2: insulin-like growth factor 2 mRNA binding protein 2; CTSH: cathepsin H; PCNA: proliferating cell nuclear antigen.

To further elucidate the oncogenic role of IGF2BP2 in TC and its interaction with CTSH, rescue experiments were conducted by overexpressing CTSH in TC cells with IGF2BP2 knockdown. It was hypothesized that CTSH might mediate some of the cancer-promoting effects of IGF2 BP2, and its overexpression could potentially reverse the effects of IGF2 BP2 knockdown. High overexpression efficiency of the pcDNA-CTSH plasmid was confirmed by RT-qPCR (**Fig 5A**). IGF2 BP2 knockdown reduced TC cell viability and downregulated PCNA expression, effects that were partially reversed by ectopic CTSH expression (Fig 5B–D). Specifically, the rescue rate for cell viability, as measured by the CCK8 assay, was 63.52% for TPC-1 cells and 76.01% for BCPAP cells (**Fig 5B**). The rescue rate for PCNA expression in TPC-1 cells was 78.21%, while in BCPAP cells, it was 69.08% (**Fig 5C**, D). These results suggest that IGF2 BP2 promotes TC cell proliferation partially through its target CTSH, offering insight into a potential mechanism by which IGF2 BP2 drives TC growth and highlighting possible therapeutic targets.

### IGF2 BP2 knockdown-mediated suppressive effects on TC cell migration, invasion, and EMT are partly overturned by CTSH overexpression

**Fig 6:**
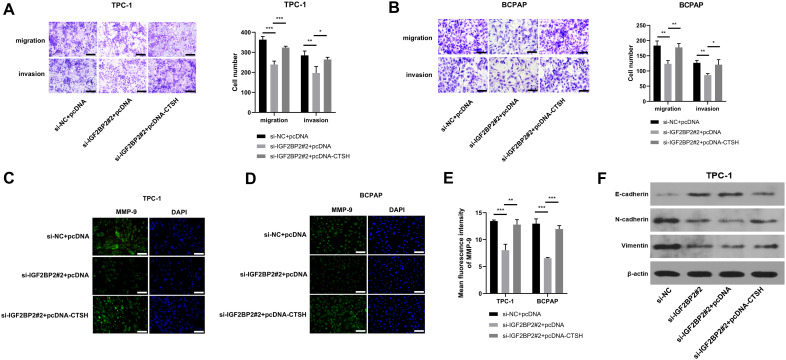
IGF2 BP2 knockdown-mediated suppression effects of TC cell migration, invasion, and EMT are partially reversed by CTSH overexpression. **(A-E)** TPC-1 and BCPAP cells were divided into three groups: si-NC + pcDNA, si-IGF2 BP2#2 + pcDNA, and si-IGF2 BP2#2 + pcDNA-CTSH. **(A and B)** Cell migration and invasion abilities were assessed by transwell assays. Scale bars: 100 μm. **(C-E)** MMP-9 protein expression was measured by IF assay. Scale bars: 100 μm. **(F)** Western blot was performed to detect the expression of EMT-associated markers (E-cadherin, N-cadherin, and Vimentin) in TPC-1 cells transfected with si-IGF2 BP2#2 alone or in combination with the pcDNA-CTSH plasmid. ^*^P < 0.05, ^**^P < 0.01, ^***^P < 0.001. IGF2 BP2: insulin-like growth factor 2 mRNA binding protein 2; CTSH: cathepsin H; MMP-9: matrix metalloproteinase 9.

IGF2 BP2 knockdown inhibited TC cell migration and invasion, critical processes in cancer metastasis, and was used to assess the metastatic potential of TC cells. CTSH overexpression partially rescued the migration and invasion capabilities of TC cells (**Fig 6A**, B). Specifically, the rescue rate for TPC-1 cell migration was 76.35%, and for invasion, it was 82.51% (**Fig 6A**). The rescue rate for BCPAP cell migration was 93.05%, and for invasion, it was 89.36% (**Fig 6B**). These results suggest that CTSH may play a compensatory role in maintaining the metastatic properties of cancer cells when IGF2 BP2 is knocked down. IGF2 BP2 knockdown reduced MMP-9 expression, and MMP-9 levels were partially restored by CTSH overexpression (Fig 6C–E), with rescue rates of 92.30% for TPC-1 cells and 91.47% for BCPAP cells. Additionally, this study examined EMT-associated markers (E-cadherin, N-cadherin, and Vimentin), which are crucial for understanding how cancer cells acquire aggressive traits such as enhanced migration and invasion. IGF2 BP2 knockdown decreased N-cadherin and Vimentin expression while increasing E-cadherin levels, with these effects partially reversed by CTSH overexpression (**Fig 6F**). These results suggest that CTSH overexpression mitigates the suppressive effect of IGF2 BP2 knockdown on EMT. Collectively, these results underscore the significant role of CTSH in modulating cellular behaviors influenced by IGF2 BP2, providing deeper insights into the modulation of metastatic pathways in TC. Taken together, these findings highlight the interdependent roles of IGF2 BP2 and CTSH in regulating TC progression.

## Discussion

m6A modification is the most prevalent epigenetic modification in eukaryotic mRNA, dynamically regulated by m6A writer, eraser, and reader proteins. IGF2 BP2, identified as an m6A reader, has been shown to regulate the progression of various cancers by post-transcriptionally modulating downstream targets [15,16]. For instance, Cui *et al*. demonstrated that IGF2 BP2 stabilized YAP mRNA in an m6A-dependent manner, thus enhancing YAP expression and its downstream target ERBB2, which contributed to colorectal cancer progression [17]. Yu *et al*. reported that IGF2 BP2 promoted lymphatic metastasis and EMT in head and neck squamous carcinoma cells by increasing the stability of Slug mRNA [18].

In our previous study, IGF2 BP2 was significantly upregulated in TC samples compared to normal tissues, where it accelerated TC progression by positively regulating HAGLR expression through an m6A-dependent mechanism [13]. This oncogenic role of IGF2 BP2 in TC aligns with earlier findings by Sa *et al*., who reported that IGF2 BP2-mediated activation of ERBB2 promoted acquired resistance to tyrosine kinase inhibitors (TKIs) in the differentiation therapy of radioiodine-refractory PTC. [19]

Building upon this understanding, several studies have highlighted IGF2 BP2’s broader role in TC *via* diverse targets and pathways. Mao *et al*. elucidated the MALAT1/miR-204/IGF2 BP2/m6A-MYC axis, where lncRNA MALAT1 upregulated IGF2 BP2 expression, enhancing MYC expression *via* m6A modification recognition, thereby facilitating TC cell proliferation and invasion [20]. Chen *et al*. further explored IGF2 BP2’s involvement in EMT by stabilizing CDH12 mRNA through its role as an m6A reader, promoting invasion and metastasis in PTC and anaplastic thyroid cancer (ATC) [21]. Additionally, Wang *et al*. demonstrated that IGF2 BP2 was significantly upregulated in PTC with lymphatic metastasis by stabilizing DPP4 through an m6A-dependent mechanism [22]. Together, these studies deepen our understanding of IGF2 BP2 as a central player in TC pathophysiology, influencing various oncogenic processes through multiple downstream pathways. Our current study further corroborates IGF2 BP2’s carcinogenic role, confirming through both *in vitro* and *in vivo* models that its knockdown suppresses TC progression by regulating CTSH in an m6A-dependent manner.

To further investigate the specificity of the IGF2 BP2/CTSH axis, comprehensive analyses were conducted using differential gene expression data, the m6A2Target database, and TCGA-THCA data. These analyses identified 219 overlapping genes, which were further evaluated. GO-CC function annotation revealed significant enrichment of extracellular matrix components, with 22 genes under this category drawing particular attention. CTSH, a member of the cathepsin family, was identified as one of these genes. CTSH is a lysosomal cysteine protease with unique aminopeptidase activity, playing a critical role in protein catabolism [23]. Extracellular cathepsins, including CTSH, have been shown to regulate cancer progression by modifying the structure of the extracellular matrix [24]. Extensive evidence has highlighted the biological roles of CTSH in cancer progression [25], cell apoptosis, [26] and neurotransmitter processing. [27] CTSH expression is frequently upregulated in several cancers, including breast cancer, [28] colorectal cancer, [29] and prostate cancer, [30] with its overexpression being strongly associated with aggressive cancer phenotypes. For instance, Jevnikar *et al*. demonstrated that CTSH-mediated processing of Talin contributed to prostate cancer progression by influencing integrin activation and adhesion strength. [30] The abnormal upregulation of CTSH facilitates cancer cell invasion through extracellular matrix degradation [31] and promotes cancer cell migration *via* activation of extracellular pathway-associated kinases. [32] Furthermore, CTSH’s carcinogenic role was validated in a mouse model, where it promoted the angiogenic switch, [25] highlighting another mechanism through which CTSH contributes to cancer progression. In line with previous reports, CTSH expression was found to be upregulated in TC, [14] consistent with our findings.

Given these compelling insights, CTSH was selected for further investigation due to its significant co-expression with IGF2 BP2 in TC, suggesting it may be a direct target. CTSH exhibited a clear co-expression pattern with IGF2 BP2 in TC, indicating that CTSH mRNA might be a potential target of IGF2 BP2. Additionally, high CTSH expression was associated with higher N stage in TC. Our results demonstrated that IGF2 BP2 stabilizes CTSH mRNA, thereby increasing both its mRNA and protein levels. Moreover, RIP-qPCR and RNA pull-down assays confirmed the interaction between IGF2 BP2 and CTSH mRNA, supporting the hypothesis that CTSH mRNA is a target of IGF2 BP2. Rescue experiments further showed that CTSH overexpression significantly reversed the effects of IGF2 BP2 knockdown on TC cell proliferation, migration, invasion, and EMT. These findings suggest that IGF2 BP2 knockdown inhibits the malignant biological behaviors of TC cells primarily by downregulating CTSH.

This study identified the IGF2 BP2/CTSH axis as a critical regulator in TC progression. However, it did not fully address other potential pathways or targets of IGF2 BP2, limiting the understanding of its complete role in cancer biology. Additionally, the translational potential of the IGF2 BP2/CTSH axis warrants further investigation to evaluate its applicability in clinical settings. Next-step strategies for validating this axis in human clinical samples should include designing biomarker studies to assess the clinical significance of IGF2 BP2 and CTSH expression in patient outcomes. Furthermore, a framework for potential clinical trials could assess therapeutic interventions aimed at modulating this pathway. These efforts are crucial for translating our findings into practical treatments, ultimately advancing more personalized and effective therapeutic strategies in TC management. However, it is important to recognize that these findings are based on specific TC cell lines, which may introduce certain limitations.

This study highlights the oncogenic role of the IGF2 BP2/CTSH axis in TC and its potential as a therapeutic target. While these findings contribute to our understanding, further exploration of alternative pathways and clinical validation is essential. Such efforts will enhance the development of targeted therapies, leading to improved treatment strategies for patients with TC.

## Materials and methods

### Data resource

Transcriptomic profiles (FPKM format) of PTC were sourced from The Cancer Genome Atlas (TCGA) portal (https://portal.gdc.cancer.gov). A cohort of 497 class A PTC specimens (tissue codes 01A/06A) were curated for statistical interrogation. Associations between IGF2 BP2 expression and tumor M, N, T stage, and tumor malignancy grade were assessed.

Concomitantly, FPKM data from the TCGA-THCA (thyroid carcinoma) repository encompassing 510 samples were procured. Correlative analyses examined relationships between cathepsin H (CTSH) expression and tumor M, N, T stage, and age.

### Immunohistochemistry (IHC) assay

Fourteen patients with PTC were enrolled in the clinical study, with ethical approval granted by The First Affiliated Hospital of Xinxiang Medical College Ethics Committee (Protocol No. EC-024–378), and their clinical information is summarized in S3 Table. All procedures complied with institutional ethical mandates (detailed in Methods). Resected PTC tissues underwent IHC processing. Additionally, xenograft tumors were subjected to IHC for Ki-67, caspase-3, and MMP-9 localization. Briefly, tissue specimens were fixed in 4% paraformaldehyde at room temperature for 24 hours, paraffin-embedded, and sectioned at 5 µm thickness. Immunostaining employed the VECTASTAIN Elite ABC kit (Vector Laboratories, USA). Six randomly captured fields (200 × magnification) per slide were digitized. ImageJ software (NIH) facilitated quantification: nuclear counterstaining identified total cells, while the “Cell Counter” plugin enumerated positively stained cells. Data were normalized as percentage of positive cells per field.

### Cell culture and transfection

Human TC cell lines, TPC-1 and BCPAP (Procell Life Science & Technology, Wuhan) were maintained in RPMI-1640 medium (Thermo Fisher Scientific) enriched with 10% fetal bovine serum (FBS; Thermo Fisher) and 1% penicillin/streptomycin (Solarbio Technology) under 37°C/5% CO_2_ conditions. Transient transfections employed Lipofectamine 2000 (Thermo Fisher) with the following siRNA constructs:

si-IGF2 BP2#1:

Sense: 5’-AGAUAGAGAUUAUGAAGAAGC-3’;

Antisense: 5’-UUCUUCAUAAUCUCUAUCUCA-3’.

si-IGF2 BP2#2:

Sense: 5’-GUUGAUUACUCAGUCUCUAAA-3’.

Antisense: 5’-UAGAGACUGAGUAAUCAACUU-3’.

An siRNA-resistant IGF2 BP2 mutant (IGF2 BP2-MUT) was engineered via silent mutations at CDS positions 303 (T → C), 309 (C → T), and 315 (C → T) within the siRNA#2 target region (coding strand: 5’-GTTGATTACTCAGTCTCTAAA-3’). The resultant sequence (5’-GTCGATTATTCAGTTTCTAAA-3’) preserves the native amino acid sequence (Val-Asp-Tyr-Ser-Val-Ser-Lys) while ablating siRNA#2 binding.

### RT-qPCR

Total RNA was isolated from TC cells using Trizol (Invitrogen) and quantified spectrophotometrically (Thermo Fisher). cDNA synthesis utilized the SweScript RT II Kit (Servicebio). Quantitative PCR reactions contained SYBR Green Master Mix (Thermo Fisher) and were processed on an Applied Biosystems instrument with amplification parameters: 95°C/10 min, 40 cycles of 95°C/15 sec and 60°C/1 min. β-actin served as endogenous control, with fold-changes calculated via 2^–ΔΔCt^. Primer sequences:

IGF2 BP2: Forward primer (F): 5’-AGGCCAGACAGATTGATTTCC-3’;

IGF2 BP2: Reverse primer (R): 5’-CGGGACTGGGTCTGCTTAG-3’.

CTSH (F): 5’-TCATGGATGTCTAAGCACCGT-3’;

CTSH (R): 5’-CCCCAGTGGTGGAGAAAGTC-3’.

β-actin (F): 5’-ACCCACACTGTGCCCATCTA-3’;

β-actin (R): 5’-GCCGTGGTGGTGAAGCTGT-3’.

### Cell counting kit-8 (CCK8) assay

Cell proliferation kinetics were interrogated using a commercial CCK8 kit (Solarbio). Transfected TC cells were seeded in 96-well plates at a density of 1 × 10^4^ cells/well with 100 μL of medium per well. At 24, 48, 72, and 96 hours post-seeding, 10 μL CCK8 reagent was introduced per well followed by 2-hours incubation. Absorbance was quantified at 450 nm.

### EdU incorporation assay

Proliferative activity was evaluated with the Click-iT EdU-555 cell proliferation detection kit (Servicebio). Transfected TC cells were re-plated in 96-well plates. After 24 hours, 100 μL EdU reagent was administered per well for 2-hours incubation. Cells then underwent fixation with 4% paraformaldehyde (30 min), permeabilization with 0.5% Triton X-100 (10 min), staining with 1 × Apollo solution (30 min), and nuclear counterstain using Hoechst 33342. The proliferative index was determined using ImageJ software with the “Cell Counter” plugin, counting the number of EdU-positive nuclei and total nuclei per field. Results are presented as the percentage of EdU-positive cells.

### IF assay

For IF staining, TC cells were fixed with 4% paraformaldehyde after 48 hours of transfection, permeabilized with 0.1% Triton solution, and blocked with 1% BSA. Primary antibodies, anti-PCNA (Cat No. GB11010; 1:500; Servicebio) and anti-MMP-9 (Cat No. GB11132; 1:200; Servicebio), were incubated overnight at 4°C. After washing three times, cells were incubated with fluorescent secondary antibody for 1 hour. Nuclei were counterstained with DAPI. Fluorescence images were acquired by confocal microscope. Mean fluorescence intensity (MFI) of target proteins was quantified across ≥6 representative fields using ImageJ with background subtraction.

### Transwell assays

Cell migration and invasion capacities were interrogated using transwell assays with routine and Matrigel-coated transwell chambers. Transfectants were digested, resuspended in serum-free medium at a density of 1 × 10^5^ cells/mL, and seeded in upper chambers (200 μL containing 2 × 10^4^ cells). Lower chambers received 650 μL complete medium. Following 24-h incubation, non-migrated/invaded were removed using a cotton swab. Migrated or invaded cells were stained with 0.1% crystal violet for 10 minutes, and the cell count from five random fields was manually recorded.

### Xenograft tumor model

A total of 10 male Balb/c nude mice (18–22 g, 5 weeks old) were purchased from Jiangsu Jicuiyaokang Biotechnology (Jiangsu, China) and housed under specific pathogen-free (SPF) conditions. After one week of acclimatization with a standard diet, the mice were randomly assigned to either the sh-NC group or the sh-IGF2 BP2 group. BCPAP cells (1 × 10^7^/200 μL) were subcutaneously injected into the mice, followed by intratumoral multi-point injections of adenovirus expressing sh-NC or sh-IGF2 BP2 every other day for 21 days, when the xenograft tumors reached approximately 100 mm^3^ in size. Tumor size was measured with a vernier caliper every 7 days, and tumor volume was calculated using the formula: tumor volume = the longest diameter × the shortest diameter^2^ × 1/2. The criteria for euthanizing the mice included reaching a tumor volume of 1500 mm^3^, a weight loss exceeding 15%, or exhibiting signs of severe distress such as impaired mobility or inability to eat or drink. When these endpoints were met, euthanasia was performed within 24 hours to prevent unnecessary suffering. No additional surgical procedures were performed that would induce significant pain, and no supplementary anesthesia was administered. Mice were euthanized at day 35 *via* intraperitoneal injection of pentobarbital sodium (150 mg/kg), and the tumors were excised and photographed. All mice survived to scheduled endpoint (day 35), whereupon tumors were excised and photographed. Daily health monitoring confirmed absence of intercurrent mortality. SPF husbandry and staff training in animal handling ensured ethical compliance and minimization of distress.

### TUNEL staining assay

Paraffin-embedded tumor sections underwent deparaffinization, rehydration, and brief centrifugation-drying. Tissue margins were circumscribed with a hydrophobic barrier pen to prevent reagent diffusion. Protease K working solution was applied to the outlined areas and incubated at 37°C for 20 minutes. The tissues were washed with PBS three times for 5 minutes each. After spin-drying, 0.1% Tween solution was added and incubated at room temperature for 20 minutes, followed by washing with PBS three times for 5 minutes each. The slices were spin-dried, and a buffer solution was added and incubated with the tissues at room temperature for 10 minutes. TDT enzyme, dUTP, and buffer from the TUNEL kit were mixed at a ratio of 1:5:50, and the solution was applied to the outlined areas. The slides were placed in a wet box and incubated at 37°C for 2 hours. After washing with PBS three times for 5 minutes each, DAPI dye solution was added and incubated at room temperature for 10 minutes, protected from light. The slides were sealed with an anti-fluorescence quenching mounting medium, observed under a fluorescence microscope, and photographed. ImageJ software was used to count TUNEL-positive and total DAPI-stained nuclei per field, and the results are presented as the percentage of TUNEL-positive cells.

### Bioinformatics analysis

IGF2 BP2 targets: Predicted via m6A2Target (http://m6a2target.canceromics.org).

Co-expression analysis: TCGA-THCA data filtered for |Pearson R| > 0.4 and *P* < 0.05.

DEG screening: |log_2_FC| > 1 and *P*adj < 0.05 (PTC vs. normal).

Functional annotation: GO analysis via DAVID (https://david.ncifcrf.gov/).

Diagnostic validation: ROC curves generated with pROC v1.18.0.

Correlation analysis: Spearman’s rank correlation for CTSH/IGF2 BP2 expression (ggplot2 v3.3.6 visualization).

### Western blot assay

Cell lysates prepared in RIPA buffer (Beyotime) underwent BCA quantification. Equal protein loads (30 μg/lane) were resolved by SDS-PAGE and electrotransferred to PVDF membranes (Millipore). Membranes were blocked with 5% skim milk (room temperature, 1 hour) then probed with primary antibodies (4°C overnight), including anti-CTSH (Cat No. 10315–1-AP; 1:800; Proteintech, Wuhan, China), anti-IGF2 BP2 (Cat No. 11601–1-AP; 1:8000; Proteintech), anti-E-cadherin (Cat No. 20874–1-AP; 1:10000; Proteintech), anti-N-cadherin (Cat No. 22018–1-AP; 1:10000; Proteintech), anti-Vimentin (Cat No. 10366–1-AP; 1:5000; Proteintech), and anti-β-actin (Cat No. ab8227; 1:20000; Abcam). The following day, membranes were brought to room temperature for 30 minutes, washed three times with 1 × TBST solution for 5 minutes each, and incubated with HRP-labeled secondary antibody at room temperature for 2 hours. The membranes were then washed again with 1 × TBST solution for 5 minutes each. Signal development employed ECL kit (Beyotime). Band densitometry quantified using ImageJ (NIH).

### Actinomycin D (ActD) assay

Transfected TC cells received 2 μg/mL actinomycin D (ActD). Cells underwent chronological sampling at 0, 1, 2, and 4 hours post-treatment. RNA isolation and RT-qPCR quantified CTSH mRNA decay kinetics across timepoints.

### RIP-qPCR assay

At 80–90% confluence, TC cells were lysed in RIP buffer. Lysates were centrifuged (14,000 rpm, 10 min, 4°C), and the supernatant was used for the following procedures. Magnetic beads were pre-treated with RIP washing buffer, and 10 μg of anti-IgG or anti-IGF2 BP2 was incubated with the beads for 30 minutes at room temperature. The supernatant was then added to the beads-antibody mixture and incubated overnight at 4°C. Post-washing, co-precipitated RNAs were extracted and resuspended in 20 μL DEPC-H_2_O. Equal RNA inputs underwent cDNA synthesis and CTSH-specific qPCR.

### RNA-pull down assay

Biotinylated RNA probes (CTSH sense/antisense control) were synthesized using the Thermo Scientific Pierce RNA 3’ Desthiobiotinylation Kit (Thermo Fisher Scientific). Streptavidin magnetic beads were saturated with 50 pmol probes at room temperature for 30 minutes. The RNA-protein binding reaction was then prepared.

**Table:** 

Reagent	Volume
50% glycerol	30 µL
10 × Protein-RNA Binding Buffer	10 µL
Cell Lysate	1-30 µL (20–200 µg)
Nuclease-free water	Up to 100 µL

Binding reaction proceeded at 4°C for 60 minutes. Washed complexes were eluted in 1 × SDS-PAGE loading buffer, denatured (100°C, 10 minutes), and subjected to IGF2 BP2 immunoblotting.

### Data analysis

Data analysis was performed using GraphPad Prism 8.0 software (GraphPad Prism, La Jolla, CA, USA), and results are presented as mean ± standard deviation (SD). All experiments were conducted in triplicate unless otherwise noted. Statistical differences were evaluated using Student’s *t*-test, one-way analysis of variance (ANOVA), or two-way ANOVA. ROC curves were plotted to assess the diagnostic value of CTSH in TC. Spearman correlation analysis was conducted to analyze the linear relationship between CTSH and IGF2 BP2 expression. A *P*-value of < 0.05 was considered statistically significant.

## Conclusion

In conclusion, IGF2 BP2 promotes TC cell proliferation, migration, and invasion by stabilizing CTSH mRNA in an m6A-dependent manner. Both IGF2 BP2 and CTSH are significantly upregulated in TC and may serve as novel diagnostic and therapeutic targets for the disease. CTSH influences cancer progression through various molecular mechanisms. Future research will investigate the downstream molecular mechanisms of the IGF2 BP2/CTSH axis in TC progression.

## Supporting information

S1 Table Gene information from the Venn diagram. (CSV)

S2 Table GO-CC analysis of overlapping genes.
(CSV)

S3 Table Clinical information of 14 patients with PTC.
(CSV)

S1 Fig The siRNA-resistant IGF2 BP2 mutant (IGF2 BP2-MUT) confirms the specificity of IGF2 BP2 knockdown. (A) RT-qPCR analysis of IGF2 BP2 mRNA levels in cells transfected with si-NC, si-IGF2 BP2#2, or si-IGF2 BP2#2 combined with empty vector (Vec), IGF2 BP2-WT, or IGF2 BP2-MUT plasmids. (B) Western blot detection of IGF2 BP2 protein expression. (C) CCK8 assay to measure proliferation at 24–96 hours post-transfection. (D) Transwell migration and invasion assays were utilized to evaluate cell migration and invasion capacities. Scale bars: 100 μm. ^*^P < 0.05, ^**^P < 0.01, ^***^P < 0.001, ns: not statistically significant. IGF2 BP2: insulin-like growth factor 2 mRNA binding protein 2; si-NC: small interfering RNA negative control; WT: wild-type; MUT: mutant; Vec: vector.
(TIF)
