# Supplementary figures and images for: IGF2BP2 contributes to thyroid cancer progression by enhancing the stability of m6A-modified CTSH mRNA
Source: PLoS One. 2025 Oct 16;20(10):e0332061. doi: 10.1371/journal.pone.0332061 (PMC12530564; doi:10.1371/journal.pone.0332061)

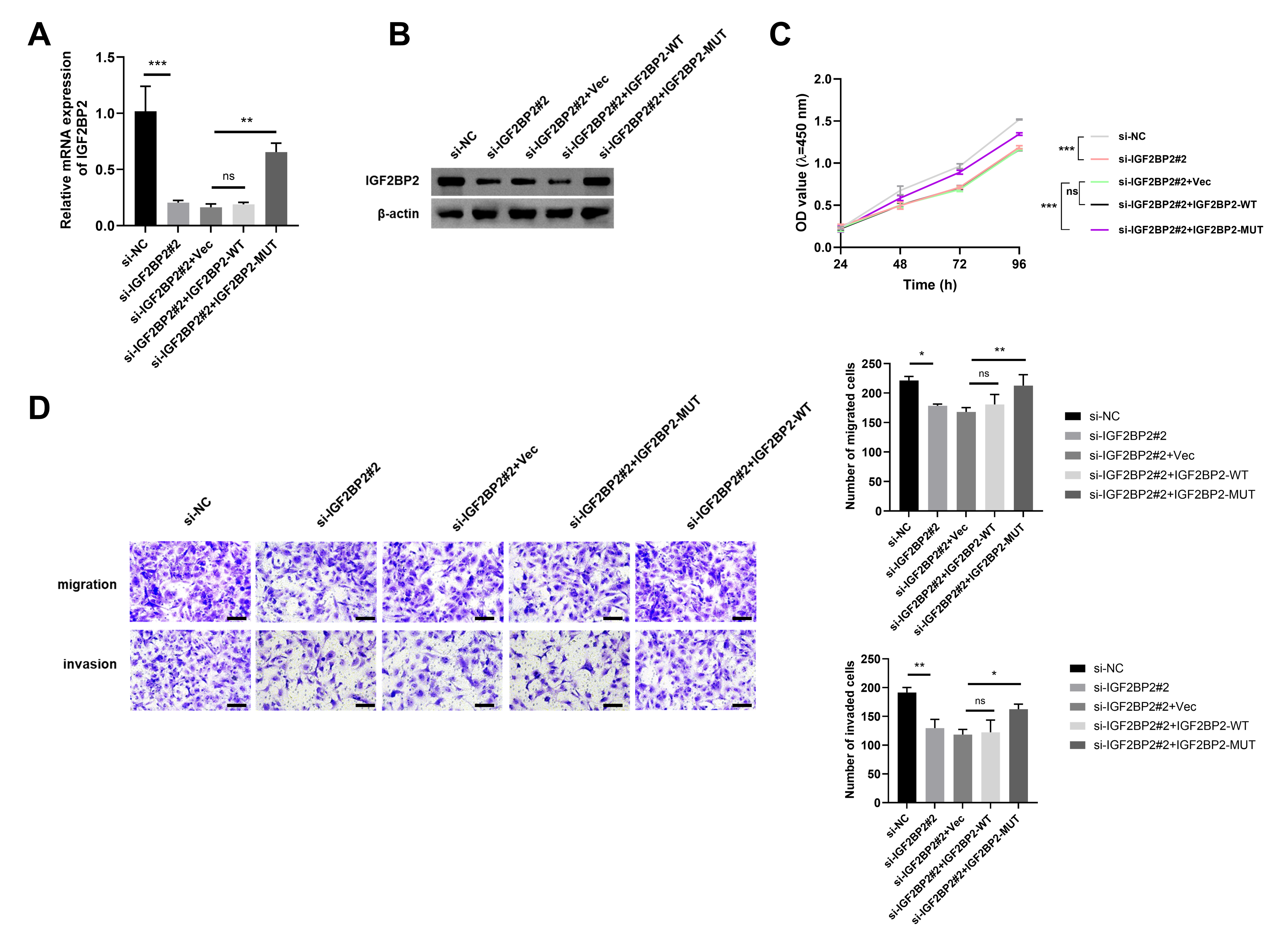

Supplement: S1 Fig — The siRNA-resistant IGF2 BP2 mutant (IGF2 BP2-MUT) confirms the specificity of IGF2 BP2 knockdown. (A) RT-qPCR analysis of IGF2 BP2 mRNA levels in cells transfected with si-NC, si-IGF2 BP2#2, or si-IGF2 BP2#2 combined with empty vector (Vec), IGF2 BP2-WT, or IGF2 BP2-MUT plasmids. (B) Western blot detection of IGF2 BP2 protein expression. (C) CCK8 assay to measure proliferation at 24–96 hours post-transfection. (D) Transwell migration and invasion assays were utilized to evaluate cell migration and invasion capacities. Scale bars: 100 μm. *P < 0.05, **P < 0.01, ***P < 0.001, ns: not statistically significant. IGF2 BP2: insulin-like growth factor 2 mRNA binding protein 2; si-NC: small interfering RNA negative control; WT: wild-type; MUT: mutant; Vec: vector. (TIF) [file pone.0332061.s004.tif]
